# Synthesis and Study of Fully Biodegradable Composites Based on Poly(butylene succinate) and Biochar

**DOI:** 10.3390/polym15041049

**Published:** 2023-02-20

**Authors:** Katerina Papadopoulou, Panagiotis A. Klonos, Apostolos Kyritsis, Ondřej Mašek, Christian Wurzer, Konstantinos Tsachouridis, Antonios D. Anastasiou, Dimitrios N. Bikiaris

**Affiliations:** 1 Laboratory of Polymer Chemistry and Technology, Department of Chemistry, Aristotle University of Thessaloniki, 54124 Thessaloniki, Greece; 2 Department of Physics, Zografou Campus, National Technical University of Athens, 15780 Athens, Greece; 3 UK BC Research Centre, School of GeoSciences, University of Edinburgh, Alexander Crum Brown Road, Edinburgh EH9 3FF, UK; 4 Department of Chemical Engineering, University of Manchester, Manchester M1 3AL, UK

**Keywords:** poly(butylene succinate), biochar, biocomposites, thermal properties, thermal conductivity, molecular mobility, mechanical properties, enzymatic hydrolysis

## Abstract

Biodegradable polymers offer a promising alternative to the global plastic problems and especially in the last decade, to the microplastics problems. For the first time, samples of poly(butylene succinate) (PBSu) biocomposites containing 1, 2.5, and 5 wt% biochar (BC) were prepared by in situ polymerization via the two-stage melt polycondensation procedure. BC was used as a filler for the PBSu to improve its mechanical properties, thermal transitions, and biodegradability. The structure of the synthesized polymers was examined by ^1^H and ^13^C nuclear magnetic resonance (NMR) and X-Ray diffraction (XRD) along with an estimation of the molecular weights, while differential scanning calorimetry (DSC) and light flash analysis (LFA) were also employed to record the thermal transitions and evaluate the thermal conductivity, respectively. It was found that the amount of BC does not affect the molecular weight of PBSu biocomposites. The fine dispersion of BC, as well as the increase in BC content in the polymeric matrix, significantly improves the tensile and impact strengths. The DSC analysis results showed that BC facilitates the crystallization of PBSu biocomposites. Due to the latter, a mild and systematic increase in thermal diffusivity and conductivity was recorded indicating that BC is a conductive material. The molecular mobility of PBSu, local and segmental, does not change significantly in the biocomposites, whereas the BC seems to cause an increase in the overall dielectric permittivity. Finally, it was found that the enzymatic hydrolysis degradation rate of biocomposites increased with the increasing BC content.

## 1. Introduction

Plastics that derive from petrochemicals, when found in the environment, do not degrade, however, they break down into smaller particles known as microplastics (typical size < 5 mm) [1]. During the last decade, microplastics became a topic of increasing concern for the environment since their concentration in water reservoirs, and even in living organisms, keeps increasing [2,3,4]. Although the health implications caused by microplastics are not clear yet, there are many efforts worldwide for the development of alternative materials which are fully biodegradable to replace the nondegradable, petroleum-based polymers [5,6]. An example of such materials are aliphatic polyesters, such as polyhydroxyalkanotes (PHA) [7], poly(lactic acid) (PLA) [8] and poly(*ε*-caprolactone) (PCL) [9], and poly(butylene succinate) (PBSu), which are intensively studied and also commercially produced [10,11]. PBSu is a semicrystalline polyester [12] and it is in high demand since it has similar physical properties to those of nonbiodegradable and commercially available polymers [13]. It has comparable characteristics such as its melting point (112–115 °C) and mechanical properties (e.g., elongation at break and tensile strength with polypropylene (PP) and low-density polyethylene (LDPE)) [10]. PBSu is produced by a polycondensation reaction between succinic acid and 1,4-butanodiol, which can be produced by the fermentation method and thus can be characterized as biobased monomers. Apart from its good physical properties, chemical resistance, and melt processability, PBSu is suitable for applications where environmental impact is a concern, e.g., agricultural, automotive, electronics, biomedical materials, packaging, etc. [10,11,13,14]. However, there are still several disadvantages related to the use of PBSu, such as the high production cost [11], high flammability [15], low melt viscosity, the low tensile and impact strength, and the relatively low biodegradability [10].

Previous studies have suggested that the use of bioadditives producing biocomposites, can help to reduce the costs and improve the biodegradability, as well as the thermal and mechanical properties, of the materials. The natural fillers of biocomposites are biomass derived from either plants or animals [16]. Such a material is BC, which is carbon-reach based and it is produced from the pyrolysis of biomass in an oxygen-limited environment at high temperatures (300–800 °C). It is an abundant and renewable material, prepared from many types of biomass, including waste and low-value feedstocks. It has received increasing attention due to its high carbon content, large specific surface area, distribution of pore size [13], hydrophobicity [17,18,19], electrical conductivity [16,19,20], and stable structure [17,20]. These physiochemical properties may vary based on the preparation conditions, like pyrolysis temperature, heating rate, particle size, and composition of feedstock [17,18,19,20]. The surface functional groups of BC (e.g., C–O, C=O, COOH, OH, etc.), and the porous surface result in an improvement in the mechanical properties of the polymer, making BC a very good reinforcing agent [16,21]. Thus, a good dispersion within a polymeric matrix containing reactive groups, in this work PBSu that contains ester, carboxyl and hydroxyl end groups, could be achieved using BC [22]. For example, in the work of Connor et al., BC with graphene nanoplatelets was used to reinforce a PBSu polymeric matrix in order to improve its thermomechanical properties [23]. Furthermore, since PBSu is semicrystalline, the reinforcing action of a filler like BC may be indirect, via the facilitation of the degree of crystallinity. Except for the latter, the semicrystalline morphology (sizes, distribution, and interconnectivity of the crystallites) alternations may induce severe effects on the macroscopic materials’ performance, thermal and electrical conductivity [24,25], and barrier properties [11]. Τhe black color and low cost of BC materials have also attracted great interest that would allow them to be used in many applications including agriculture, automotive [26], and electronics [27].

The purpose of the present work is the synthesis and study of fully biodegradable PBSu/BC biocomposites. The materials were produced for the first time via the in situ polymerization method, aiming at the improvement of the thermal and mechanical properties of PBSu. The in situ polymerization gives stronger interactions between the fillers and the polymeric phase, and thus a more uniform dispersion of the filler can be achieved, compared with the method of melt mixing [28].

## 2. Materials and Methods

### 2.1. Materials

Succinic acid (SA) (purum 99+%), and titanium isopropoxide (≥97%) (Tis) catalyst of an analytical grade were obtained from Sigma–Aldrich Chemical Co (Saint Louis, MO, USA). 1,4-Butanediol (BD) (Purity: >99%) and were purchased from Alfa Aesar (Kandel, Germany). The BC used in this work (MSP 700) was produced by intermediate pyrolysis of pelleted miscanthus straw at 700 °C in a pilot-scale rotary kiln at the UK BC Research Centre. It was dried overnight in the oven at 80 °C under a vacuum before every single use. All other reagents were of analytical grade.

### 2.2. Synthesis of PBSu Biocomposites

Poly(butylene succinate) (PBSu) was synthesized by the two-stage melt polycondensation method (esterification and polycondensation) using SA and BD in a molar ratio of 1/1.1, which were charged into a glass batch reactor. The polymerization mixture was heated at 170 °C under the presence of nitrogen for 1 h and a stirring speed of 500 rpm, at 180 °C for an additional 1 h, and finally at 190 °C for 1.5 h. The first step of the esterification procedure was completed when all the theoretical amounts of H_2_O had been distilled. In the second reaction stage of polycondensation, the vacuum was slowly increased to 5.0 Pa over a time of about 30 min, in order to avoid excessive foaming, remove the excess diol or remaining H_2_O, and, furthermore, to minimize oligomer sublimation. During this interval, the temperature was gradually increased up to 230 °C, while the stirring speed was also increased to 720 rpm. At the end of this stage, 400 ppm of Tis catalyst was added to the reactor. The reaction was maintained for 30 min at this temperature and then was increased by 10 °C every 30 min until reaching 250 °C, where the reaction was continued for 1.5 h (total polycondensation time 2.5 h). 

In a similar way, PBSu/BC biocomposites have been produced by in situ polymerization containing 1, 2.5, and 5 wt% BC. To produce a uniform and fine filler dispersion, BC was first added in BD and dispersed with ultrasonication for 5 min. Thereafter, the dispersion was introduced with SA in the glass batch reactor for the polymerization procedure to take place as described previously for neat PBSu.

### 2.3. Intrinsic Viscosity Measurement

Intrinsic viscosity [*η*] measurements were carried out using an Ubbelohde viscometer (Schott Gerate GMBH, Hofheim, Germany) at 25 °C in chloroform. In order to remove any nonsoluble materials, the solutions were filtered through a disposable membrane filter (Teflon). The average value was calculated after three different measurements. The intrinsic viscosity value of the polymer was calculated by the Solomon-Ciuta Equation (1):

(1) [η]=[2{tt0−ln(tt0)−1}]12c

where *c* is the solution concentration, *t* is the flow time of the solution and *t*_0_ is the flow time of pure solvent.

### 2.4. Gel Permeation Chromatography–Size Exclusion Chromatography (GPC/SEC)

The molecular weight of the materials was determined using gel permeation chromatography–size exclusion chromatography (GPC/SEC) analysis. An Agilent 1260 Infinity II LC system (Agilent Technologies, Santa Clara, CA, USA) equipped with an isocratic G7110B pump, an automatic vialsampler G7129A, a Refractive Index Detector (RID) G7162A and a PLgel 5 μm (50 × 7.5 mm) guard column combined with 2 PLgel 5 μm (300 × 7.5 mm) MIXED-C columns were employed for the investigation of the molecular weights. For the calibration curve, 3 poly(methyl methacrylate) (PMMA) standards of molecular weights between 0.535 and 1591 kg/mol were facilitated. The prepared solutions had a concentration of 3 mg/mL and were filtered through 0.45 μm pore size PTFE filters. The injection volume was 20 μL and the total elution time of each sample was 30 min. The temperature of the columns and the RID were both set at 40 °C.

### 2.5. Nuclear Magnetic Resonance (NMR)

NMR spectra were recorded in deuterated chloroform using an Agilent 500 spectrometer (Agilent Technologies, Santa Clara, CA, USA) for the structural study of the biocomposites at room temperature. Spectra were calibrated using the residual solvent peaks.

### 2.6. X-ray Diffraction Patterns (XRD)

XRD measurements were performed using a MiniFlex II XRD system (Rigaku Co, Tokyo, Japan) in the angle 2*θ* range from 5° to 45° with a scanning speed of 1°/min with CuK_α_ radiation (λ = 0.154 nm). All samples were in the form of films.

### 2.7. Differential Scanning Calorimetry (DSC)

The thermal transitions of PBSu, glass transition, crystallization, and melting were assessed in a nitrogen atmosphere of high purity (99.9995%), in the temperature range of −110 to 150 °C. The measurements were performed by employing a TA Q200 DSC calorimeter (TA Instruments, New Castle, DE, USA), calibrated with Indium for temperature and enthalpy and with sapphires for heat capacity. Pieces of ~8 mg in mass were cut from the prepared samples and were closed in Tzero aluminum TA pans. The first heating scan up to 150 °C was performed in order to erase the sample’s thermal history. Subsequently, the sample was cooled from 150 °C (melt state) down to −110 °C at 20 K/min, stayed there for 2 min, and, finally, heated to 150 °C at 10 K/min.

### 2.8. Polarized Light Microscope (PLM)

PLM technique was performed using a Nikon Optiphot-1 polarizing microscope (Nikon, Optiphot-2, Tokyo, Japan) equipped with a Linkam THMS 600 heated stage, a Linkam TP91 control unit, and a Jenoptic ProgRes C10Plus camera This technique was employed to assess the semicrystalline morphology (i.e., the number, size, and distributions of PBSu spherulites). PLM images were recorded during the isothermal crystallization at 90 °C of initially melted samples was recorded PLM images.

### 2.9. Light Flash Analysis (LFA)

The thermal diffusivity (*α*), and thermal conductivity (*λ*), were measured employing the light flash analysis (LFA) technique [29] by means of a NETZSCH LFA 467 HyperFlash apparatus (NETZSCH, Selb, Germany). The measurements were performed in a nitrogen atmosphere on the samples as received and cut from the produced sheets in the cylindrical form of 12.7 mm (0.5 inch) in diameter and ~1.4–1.7 mm in thickness (disk height). Prior to the measurements, graphite was spray coated onto the top and bottom sides of the samples following the manufacturer’s instructions. LFA measurements were performed at temperatures 25, 35, 45, 55, 65, and 75 °C. 

The thermal diffusivity was directly measured from the heat transmission, over an average of 3 light pulse shots of 600 μs in duration and 3 samples for each composition, at a spot size of 8.9 mm via an InSb infrared detector. A Cowan plus pulse-correction model [30] was fitted to the detectors’ signal and *α* was estimated according to the so-called ‘*half-time*’ method by Parker, using Equation (2),

𝛼 = 1.38⋅𝐿^2^/(𝜋⋅𝑡_1/2_), (2)

wherein, *t*_1/2_ is the required time for reaching the half of maximum temperature (detector signal) rise of the rear surface and *L* is the sample thickness [29]. Then, thermal conductivity was calculated via Equation (3),

𝜆 = 𝛼⋅𝜌⋅𝑐_𝑝_, (3)

wherein, *ρ* is the sample density and *c_p_* is the specific heat for a given temperature. In our case, *ρ* was estimated from accurate measurements of the sample mass and volume, whereas *c_p_* was calculated directly from LFA software, by comparison with a reference sample (Pyroceram), as suggested by the manufacturer.

### 2.10. Broadband Dielectric Spectroscopy (BDS)

The molecular mobility was studied by the advanced technique of BDS [31], employing a Novocontrol BDS setup (Novocontrol, Montabaur, GmbH, Germany), and an Alpha frequency response analyzer combined with a Quatro liquid nitrogen cryosystem. The measurements were performed in a nitrogen atmosphere and the samples had the form of sandwich-like capacitors (14 mm in diameter). Pieces of the samples were placed between finely polished brash disk-electrodes, melted at ~150 °C using thin silica spacers (~50 μm) to keep the distance between the electrodes constant and prevent electrical contact and then rapidly cooled. For this work, we recorded and evaluated the imaginary part of dielectric permittivity, *ε*″, related to the dielectric losses, as a function of frequency in the range from 10^−1^ to 10^6^ Hz, and in the temperature range between −150 and 60 °C, upon heating at steps of 5 and 10 K.

### 2.11. Mechanical Properties

Mechanical properties of the biocomposites were studied using an Instron 3344 dynamometer (Norwood, MA, USA), in accordance with ASTM D638 using a crosshead speed of 50 mm/min. Dumbbell-shaped tensile test specimens (central portions 5 × 0.7 cm thick and 1.5 cm gauge length) were prepared by compression molding in a thermopress at 160 °C, cooled rapidly, and cut in a Wallace cutting press. 

A Tinius Olsen apparatus (Horsham, PA, USA) was used under ASTM D256 for the notched Izod impact tests. In a similar way, as described before, the specimens were prepared. The samples were conditioned at 25 °C in a 50 ± 5% relative humidity environment for 48 h, before each measurement. 

The results for each sample present the average values of at least five tests. The mean values of tensile strength at yield and breakpoint, elongation at break, Young’s modulus, and impact strength were determined.

### 2.12. Scanning Electron Microscopy

SEM was carried out using a JEOL JMS 760F (Jeol, Freising, Germany) scanning microscope operating at 10–20 kV equipped with an energy-dispersive X-ray Oxford ISIS 300 microanalytical system. For this work, surfaces of thin films as well as fractured surfaces were studied. Regarding sample preparation, all surfaces were coated with carbon black in order to avoid charging under the electron beam.

### 2.13. Transmission Electron Microscopy (TEM)

TEM studies were performed with an FEI Tecnai G2 20 microscope (FEI, Hillsboro, OR, USA) at the accelerating voltage of 200 kV. Regarding sample preparation, films of neat PBSu and its biocomposites were cut with an ultramicrotome to films of 80 nm thickness (DiATOME 45° diamond knife, DiATOME Ltd., Nidau, Switzerland). The thin sections floating on the knife’s water surface were deposited on carbon-coated grids and air-dried overnight.

### 2.14. Color Measurement

Color measurements were performed using a Datacolor Spectraflash SF600 plus CT UV reflectance colorimeter (Datacolor, Marl, Germany) using the D65 illuminant, 10◦ standard observer with UV component excluded and specular component included.

The color of the biocomposites was investigated according to the CIEL*a*b* color system. The L* axis measures luminosity or lightness ranging from 0 (black) to 100 (white), the a* coordinate measures redness, when a is positive or greenness, when a is negative, and the b* coordinate measures yellowness, when b is positive or blueness, when b is negative, C* represents chroma and H* represents hue angle. The K/S fraction was calculated in order to assess the concentration of the color on the biocomposites [32].

### 2.15. Enzymatic Hydrolysis

All samples in the form of films (25 μm) were prepared using an OttoWeber Type PW 30 hydraulic press (Wuppertal, Germany). The films were placed in test tubes, wherein was 10 mL of phosphate buffer solution (0.2 M, pH 7.2 ± 0.1) with 0.09 mg/mL Rhizopus delemar lipase and 0.01 mg/mL Pseudomonas cepacia lipase were added. The test tubes were placed in the oven at 50 ± 1 °C for 1 month and the buffer/enzymes solution was replaced every 3 days. After a specific time of 5, 10, 15, 20, 25, and 30 days, the films were removed from the buffer/enzymes solution, washed thoroughly with distilled water, and then dried at room temperature under a vacuum, till constant weight. Every measurement was repeated three times. The rate of enzymatic hydrolysis was estimated from mass loss. Alterations of the morphology of the films after enzymatic hydrolysis were examined using SEM JEOL (JMS 760F).

## 3. Results

### 3.1. BC Synthesis and Characterization

**Figure 1:**
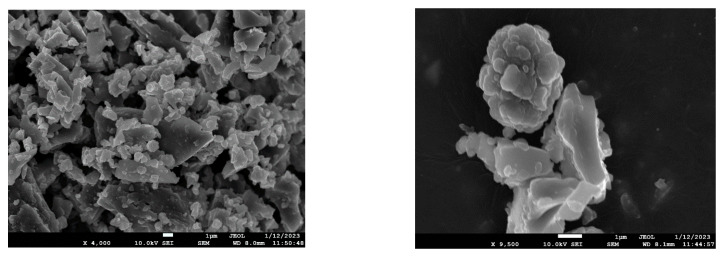
SEM micrographs of prepared BC after miscanthus pyrolysis at 700 °C and grinding in low (**left**) and high (**right**) magnification.

While the as produced BC retained the shape of the original pellets, grinding the BC to the appropriate size for the experiments yielded irregular particle shapes and sizes (Figure 1). From SEM micrographs, both large particles with sizes higher than 10 μm (10–15 μm), and also much smaller particles with sizes at the nanoscale level (100–200 nm), can be detected.

### 3.2. Synthesis and Characterization of In Situ Prepared PBSu/BC Biocomposites

PBSu biocomposites were synthesized by two-step polycondensation reactions of esterification and polycondensation. According to this procedure, pristine PBSu and its biocomposites with high molecular weights were prepared. The *M*n (number average molecular weight) and *M*w (weight average molecular weight) values of neat PBSu are calculated as 70,350 g/mol and 117,800 g/mol, respectively, which are very satisfying for aliphatic polyesters. Very close to that on neat PBSu are also the intrinsic viscosities and molecular weights of PBSu/BC biocomposites (Table 1). All of the biocomposite’s *M*n is in the range of about 62,000–67,000 g/mol without any clear effect of the BC amount on the molecular weight of PBSu. Usually, when additives have some reactive groups, they can interact with the polymer matrix, affecting its molecular weight.

**Scheme 1:**
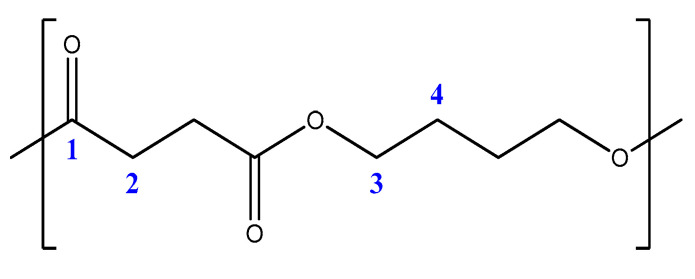
Numbered structures of the repeating units of the prepared PBSu and PBSu/BC biocomposites.

**Figure 2:**
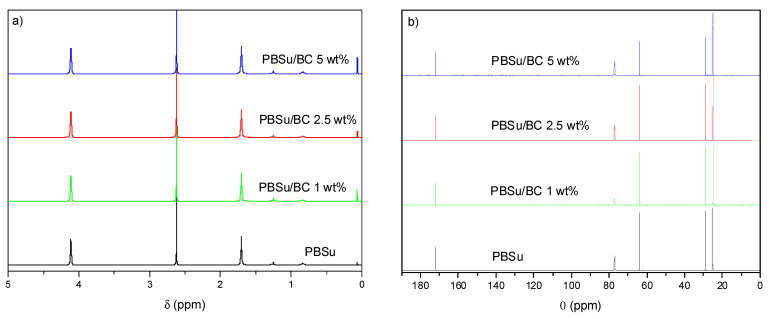
(**a**) ^1^H NMR and (**b**) ^13^C NMR spectra of the prepared biocomposites.

To evaluate the successful synthesis of the PBSu and its biocomposites, NMR spectra were obtained. The chemical structure of the produced aliphatic polyesters was assessed by proton (^1^H) and carbon (^13^C) nuclear magnetic resonance spectroscopy. Scheme 1 presents the numbered structures of the studied polymers. The part of the structure corresponding to carbons 1–4 appears basically at the same shifts for all samples and the assigned chemical shift in ^1^H and ^13^C remain at approximately the same positions (Figure 2). The ^1^H-NMR spectra of PBSu and its biocomposites are shown in Figure 2a. The ^1^H-NMR resonance signal of pure PBSu appears at 2.62 ppm and gives a single peak, which is associated with the methylene protons (2) in the succinic moiety. The resonances at 1.71 and 4.11 ppm are respectively assigned to the methylene protons (4) and (3) in the 1,4-butanediol unit. Figure 2b depicts the ^13^C NMR spectra of the synthesized polymers. As mentioned above, the peaks of all samples corresponding to carbons 1–4 remain basically the same. At 172.2 ppm, the deprotected carbons of the carbonyls (1) are recorded, and at 28.9 ppm, the carbons (2) of the methylene groups. The carbons that are adjacent to the oxygen atom of the ester bond (3) give a peak at 64.1 ppm, while the other methylene carbons of the aliphatic chains (4) appear at 25.1 ppm. Thus, the NMR spectra demonstrated successful synthesis of the PBSu and PBSu/BC biocomposites. The results are in accordance with the literature [33,34].

**Figure 3:**
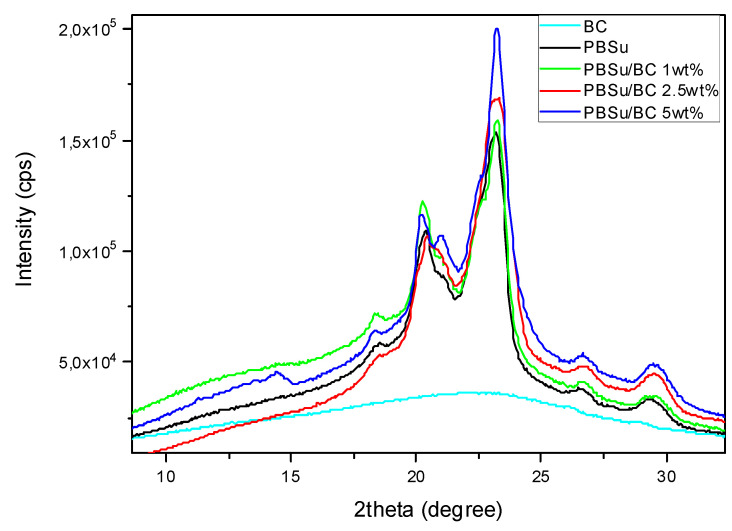
XRD patterns of neat PBSu, BC, and PBSu/BC biocomposites containing 1, 2.5, and 5 wt% of BC filler.

The XRD pattern of PBSu and its biocomposites is presented in Figure 3. The unit cell of the *α*-form of PBSu crystals is monoclinic, and the diffraction peaks of the crystal planes (020), (021), and (110) are shown in Figure 3 at angles 2*θ* = 20.1°, 21.3°, and 23.2°, respectively, which are in accordance with the literature [35,36]. These peaks are also observed in biocomposites while BC is completely amorphous. The addition, the BC does not affect the crystal form of PBSu. No shift of the PBSu peaks is observed, indicating that the unit cells of the polyester and the BC remain the same. Most probably, the same stands also for the density of the crystals and the chain packing.

### 3.3. Thermal Transitions 

**Figure 4:**
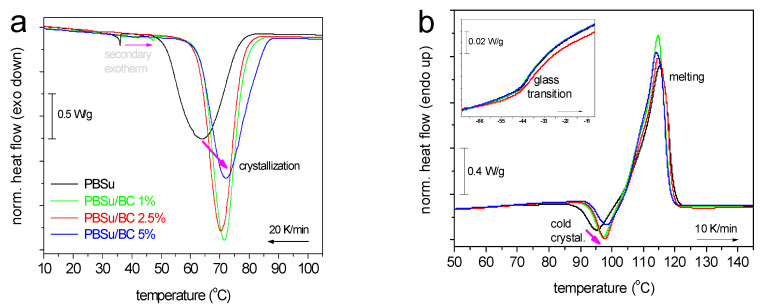
Comparative DSC traces for all samples during (**a**) cooling from the melt state and (**b**) subsequent heating. The added arrows mark the effects of the BC addition to the crystallization events. The inset to (**b**) shows a focus on the glass transition region.

To assess the direct BC effects on crystallization (e.g., nucleation) and glass transition (interfacial interactions), an attempt was made to eliminate crystallization during cooling by melting and subsequent fast cooling (>100 K/min). However, this ‘quenching’ was not sufficient to vanish crystallization (not shown data), as usually happens with PBSu and conventional methods of cooling in DSC [12,37]. Figure 4 presents the DSC thermograms comparatively for all compositions, upon the erasing of any thermal history (via melting), cooling at 20 K/min (Figure 4a), and subsequent heating at 10 K/min (Figure 4b). Regarding pure PBSu, the main thermal events are in accordance with previous works from the literature [12].

During cooling, PBSu exhibits an exothermal peak at 64 °C which corresponds to the formation of crystals. The addition of BC leads to an elevation of the characteristic crystallisation temperature, *T*_c_, from 64 °C (PBSu) to 70–72 °C, as well as of the crystalline fraction, CF_c_, from 30% to 33–35%. The effects of such fillers facilitating both the rate and the amount of crystallization are widely explained via the additional contribution of the fillers as nucleating agents [38,39]. A similar effect is recorded within the cold crystallization recorded upon heating in Figure 4b (*T*_cc_ in Table 2). The addition of BC was found to not affect the melting point, *T*_m_~115 °C (Table 2), which denotes quite similar quality/density of the crystal structure (lamellae thickness). 

As it is presented in Figure 4b, the glass transition temperatures were almost the same between the different samples (−35 and −34 °C). The recorded change in heat capacity, Δ*c_p_*~−0.23–0.24 J/g∙K (Table 2) is also stable. It is worthy to report that the *T*_g_ of poly(*n*-alkylene succinates) can be affected by the density of the semicrystalline morphology. For example, in the case of the formation of many tiny crystals, the amorphous polymer chain located between the crystals may suffer severe spatial confinement. In such a case, the *T*_g_ may drop significantly [12]. 

### 3.4. PLM Results

**Figure 5:**
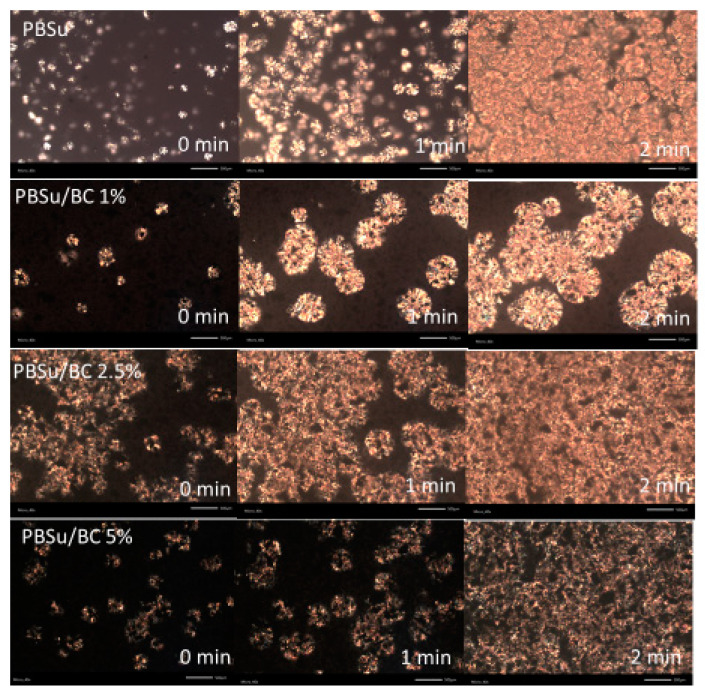
Polarized light microscopy (PLM) images during the time evolution of isothermal crystallization of all samples at 90 °C. The added scale bars correspond to 500 μm and magnification micro-40.

PLM measurements were performed during the evolution of the same isothermal annealing at 90 °C to record the differences in the size and population of the formed spherulites. In Figure 5, the initial, intermediate, and final states of crystallization (0 min, 1 min, and 2 min) are presented. It is clear that the size of spherulites increased in the biocomposites. The number of crystals is high for undoped PBSu and for 2.5 wt% BC, while is significantly less for 1 and 5 wt%. This may suggest that the nucleating activity of BC, and the particle distribution throughout the PBSu matrix, are optimized at 2.5 wt% BC. In all cases of biocomposites, the spherulitic growth rate is much larger as compared to the unfilled PBSu. The latter may originate from easier chains diffusion and/or better folding capability of the PBSu chains in the composites. 

### 3.5. Thermal Diffusivity/Conductivity

**Figure 6:**
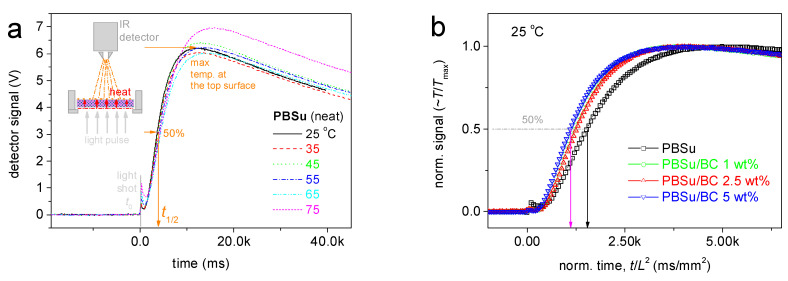
(**a**) Typical LFA measurements for neat PBSu at various temperatures. The added arrows describe the route for estimation of t_1/2_ (to be used for the calculation of thermal diffusivity). (**b**) Comparative LFA results for all samples at 25 °C, in terms of (vertical axis) normalized signal, namely, signal/signal max, corresponding to T/T_max_ at the top of the sample surface, and (horizontal axis) normalized time, i.e., t/L^2^, where L is the sample thickness.

Figure 6a presents the raw results of the LFA technique for pristine PBSu and for the overall temperature range studied. In Figure 6b, similar results are shown comparatively for all systems at 25 °C. To compare the results between the different samples, we have normalized both the measurement time, *t*, as *t*/*L*^2^, and the detector signal, *T*, as *T*/*T*_max_. As shown in Figure 6b, the biocomposites exhibit faster heat transfer, and, thus, increased thermal diffusivity as compared to the unfilled PBSu.

**Figure 7:**
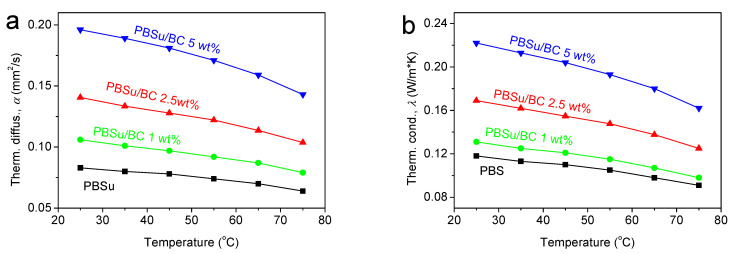
The temperature dependence of (**a**) thermal diffusivity, *α*, and (**b**) thermal conductivity, *λ*, for all samples (compositions).

Employing the Parker Equation (2), *α* values were estimated. Also, employing these values along with the measured heat capacity, *λ* values were estimated according to Equation (3). The corresponding values are listed in Table 3 and shown in Figure 7 as a function of temperature and BC loading.

For neat PBSu, *α* and *λ* equal 0.08 mm^2^/s and 0.12 W/m∙K, respectively. Both values exhibit an increase in the presence of BC, up to 0.20 mm^2^/s and 0.22 W/m∙K respectively. Interestingly, the increase observed for *α* and *λ* is monotonic with the BC loading. These values of thermal conductivity seem low as compared to the highly conductive materials (e.g., metals, carbon nanotubes) [40], whereas they are typical for polymers. In the case of amorphous polymers, it is believed that the presence of thermally conducting fillers that do not percolate [25], as expected here, may lead to a drop in *λ*. This is due to the so-called ‘interfacial thermal resistance’ effect as the filler interfaces facilitate heat scattering. On the other hand, when compared to an amorphous polymer, the presence of crystals of the same polymer may increase thermal conductivity [40]. This is compatible with our case as PBSu is a semicrystalline polymer. Therefore, it will be checked in the following as to whether the BC effects in *α* and *λ* are either direct or indirect via alternations in crystallization.

**Figure 8:**
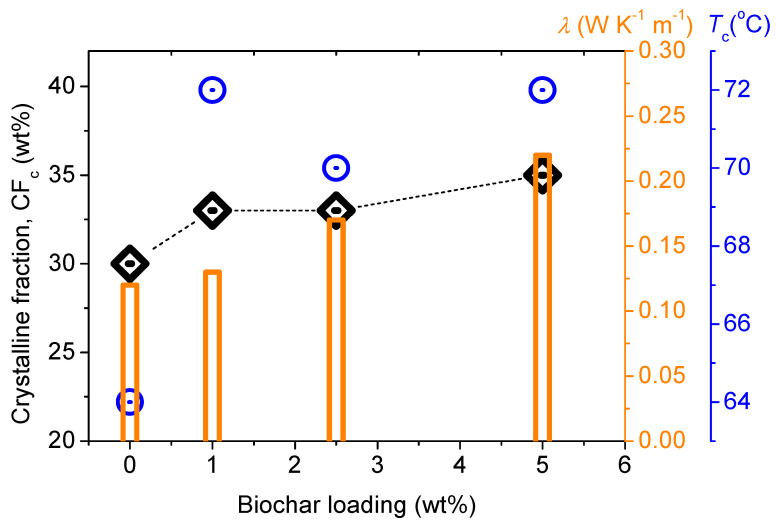
Effects of composition on (left axis, black diamonds) crystalline fraction, comparatively with (right axis, blue circles) crystallization temperature and (orange columns) thermal conductivity (LFA).

It is useful, at this point, to directly compare CF_c_ with the recorded increase in thermal conductivity (Figure 8) Both values were found to increase monotonically with BC. Considering the low absolute *λ* values, it can be concluded that any improvement in heat transport is due to the increasing number of percolating PBSu spherulite paths. It is known that the main heat transport carriers are the phonons [25,41], the number of which is enhanced when the ‘ordered’ structure/entities increase. This happens with the crystalline (ordered) as compared to the amorphous (disordered) phases.

### 3.6. Molecular Dynamics 

**Figure 9:**
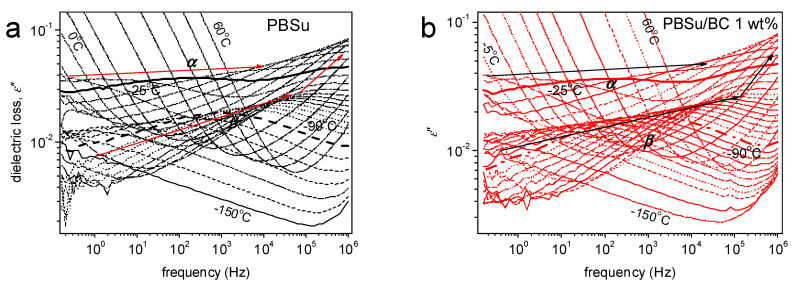

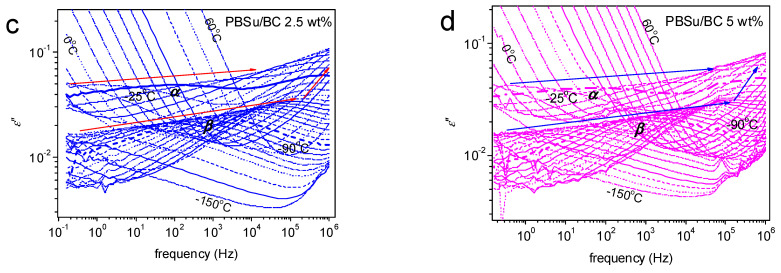
Comparative isothermal curves of the imaginary part of dielectric permittivity, ε″ (dielectric loss), against frequency for all samples indicated on the plots and temperatures. The evolution of the local β and segmental α relaxations are marked by the added arrows.

The molecular dynamics were assessed by BDS, via effects on the imaginary part of dielectric permittivity, *ε*″. The latter is related to the dielectric losses [41]. The initial recordings, namely the isothermal *ε*″(*f*) spectra, were obtained at various temperatures from −150 °C up to 60 °C on samples initially melted (erased thermal history) and subsequently cooled at ~10 K/min. This sets the results comparable to those of the DSC, regarding the semicrystalline state (CF_c_) and the *T*_g_ (Figure 9).

**Figure 10:**
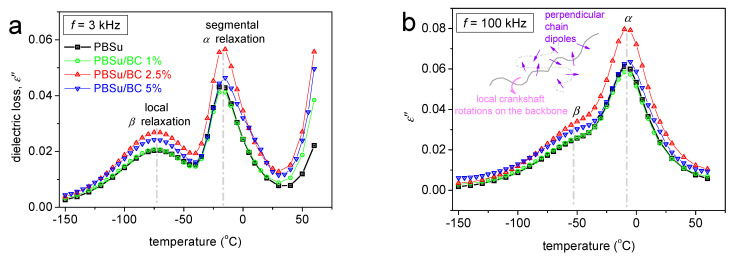
Comparative isochronal BDS curves, namely, the temperature dependence of the imaginary part of dielectric permittivity, *ε*″, for PBSu and the PBSu/BC biocomposites, for (**a**) *f* = 3 kHz and (**b**) *f* = 100 kHz. Indicated are the local and dielectric relaxation processes.

The molecular dipolar relaxations are followed in these data as ‘peaks’ of the *ε*″(*f*), the maxima of which migrated at gradually increasing frequencies, *f*_max_, with the increase in temperature. This was due to the simultaneous lowering of the relaxation times (*τ*_rel_) of the corresponding molecular groups/chains. Two types of mobilities (peaks) are recorded for all samples, i.e., the local *β* process at the lower temperatures (*T* < *T*_g_) and the stronger segmental *α* process at *T* ≥ *T*_g_. To employ a more direct comparison with calorimetry, we replotted the isothermal data of Figure 9 and show them as comparative isochronal plots of *ε*″ against temperature in Figure 10.

The local *β* process has been assigned to the crankshaft rotation of the ester group (dipolar) in the backbone of the polymers [12,42]. Then, the *α* process is considered to arise from dipoles perpendicular to the polymer chains. Thus, it is coupled to segmental chain motions and is called ‘glass transition dynamics’. 

**Figure 11:**
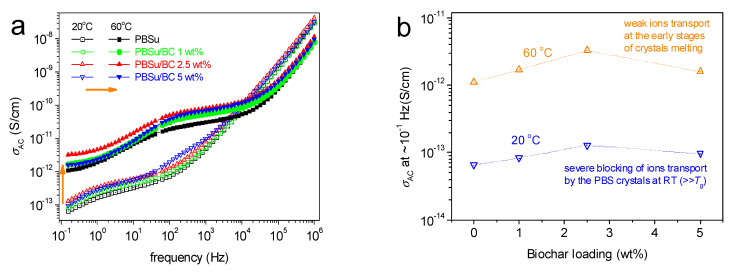
(**a**) Comparative isothermals of the AC conductivity, *σ*_AC_, for all samples at 20 and 60 °C. (**b**) The BC loading dependence of *σ*_AC_ at the lower frequency of measurement.

The introduction of BC does not seem to induce worth-noting alternations in molecular dynamics, as the processes barely change position (*f*_max_/*T*_max_ in Figure 9 and Figure 10). This suggests no strong interactions between the polymer and the BC that could hinder both relaxations, which is the expected case when the ‘filler’ offers crystallization nucleation sites [43]. However, we always need to keep in mind that all the tested systems concern semicrystalline materials and, therefore, any effects of BC are indirect (via crystallinity). There was recorded, nevertheless, an increase in the overall dielectric signal (Figure 10) of PBSu in the biocomposites, even at quite lower temperatures. This suggests that BC induces an overall increase in dielectric permittivity (real part, *ε*′). Further analysis of the results, e.g., in terms of electrical conductivity, *σ*_AC_, (at RT and higher temperatures, Figure 11) do not show tremendous effects by BC.

The PBSu/BC systems seem to be electrically insulating materials, similar to the neat polymer. This is true at least up to ~50 °C, i.e., highly above *T*_g_. In Figure 11**,** *σ*_AC_ at high temperatures tends to demonstrate a plateau at the lowest frequencies. This is due to ionic conductivity, being relatively low (10^−12^−10^−11^ S/cm). The ions transport throughout the sample volume is most probably blocked by the polymer crystals. Thus, at higher temperatures (e.g., 60 °C), more amorphous polymer paths are formed which facilitate the ions’ transport.

### 3.7. Mechanical Properties

**Figure 12:**
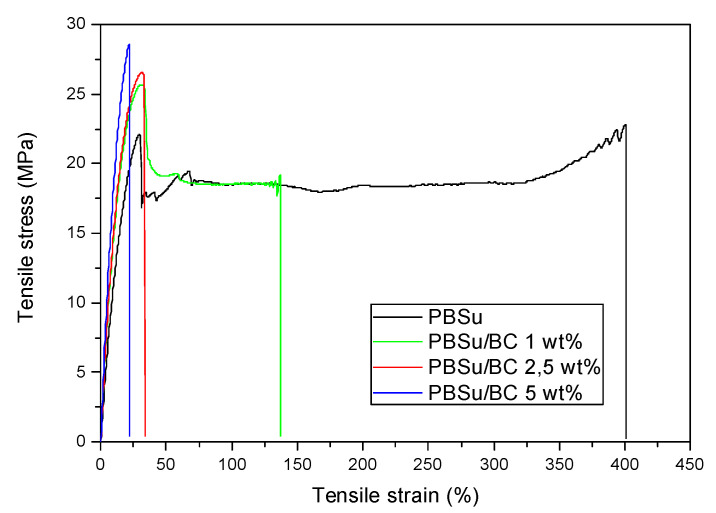
Stress–strain curves for neat PBSu and PBSu/BC biocomposites.

The results of the tensile properties of PBSu/BC composites with different BC loading levels are observed in Figure 12. From the stress–strain curves of neat PBSu and its biocomposites, it was found that PBSu is a polyester with a tensile strength of about 22.2 ± 0.5 MPa and a high elongation at break (about 440%) making it ideal for formulation in films for several applications, such as in agriculture. The mechanical behavior of the materials changed with the addition of BC (Table 4). It appears that the tensile strength at yield of the PBSu/BC composites increased with the addition of BC content filler from 22 to 26 MPa. The measurement of tensile strength at yield of PBSu/BC 5 wt% biocomposite could not be performed due to the brittleness of the corresponding specimen. The tensile strength at the break of the PBSu/BC biocomposites increased from 21 to 29 MPa, indicating that BC can act as a reinforcement agent. The dispersion, the particle size, and the aspect ratio of the BC are the main factors causing the increase in tensile strength both at yield and at the break. [44]. Similar results have been found in PLA/BC composites. Qian, et al. blended PLA with ultrafine bamboo char to improve the thermal and mechanical properties of the composites [45]. It was reported that tensile strength and modulus increased with increasing BC loadings from 5% to 30%. Zouari, et al. have prepared biocomposites using PLA, BC, and hemp fibers [19]. The results showed that upon adding 5 wt% BC, the tensile strength was increased, while Arrigo et al. reported that tensile modulus values increase with increasing BC loading in PLA/BC composites, prepared by melt mixing or solvent evaporation [46]. This increase confirms the homogeneous dispersion of the filler within the polymer matrix. Hence, the distribution, the shape, and the uniform particle sizes of BC could cause the higher tensile strength of the biocomposites. In contrast, the elongation at break for the composites decreased from 440% to 20% as the filler content was increased. A similar trend to that of tensile strength shows Young’s modulus for the biocomposites. Young’s modulus increased continuously for the biocomposites as the BC loading level increased. This is attributed to the increase in the biocomposite’s stiffness.

Impact strength analysis of the biocomposites is almost similar to the trend of its tensile strength. Table 4 shows an increase in impact strength when adding the filler. The maximum impact strength was observed for PBSu/BC 2.5 wt% composite with 1.74 J/m^2^. Lower impact strength was observed for PBSu/BC 5 wt%, with 1.41 J/m^2^., perhaps due to the brittleness of the material. The addition of BC to PBSu increases the impact strength, due to the BC filler providing a good dispersion in the polymer matrix and, also, because BC absorbs more energy and the biocomposites resist crack propagation. The results are in accordance with the work of Sundarakannan, et al. [47].

**Figure 13:**
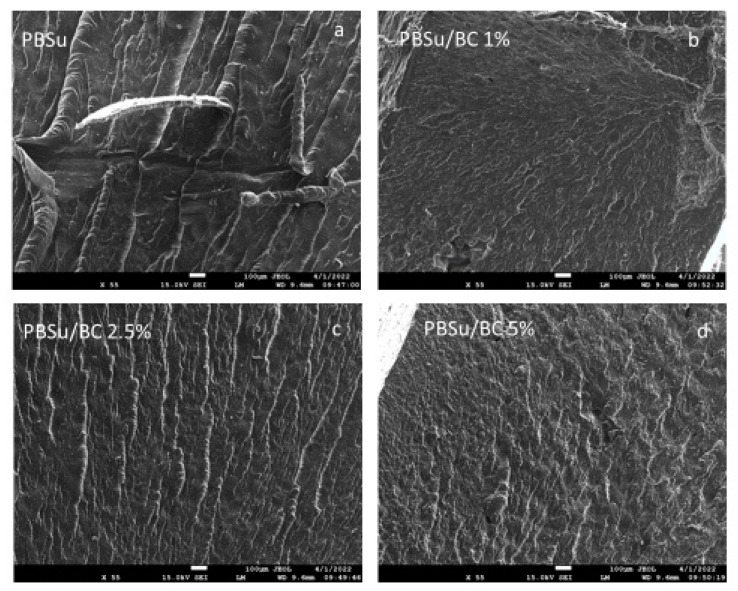
SEM micrographs of the impact fracture surfaces of PBSu/BC composite in low magnification, for samples indicated on the figure.

**Figure 14:**
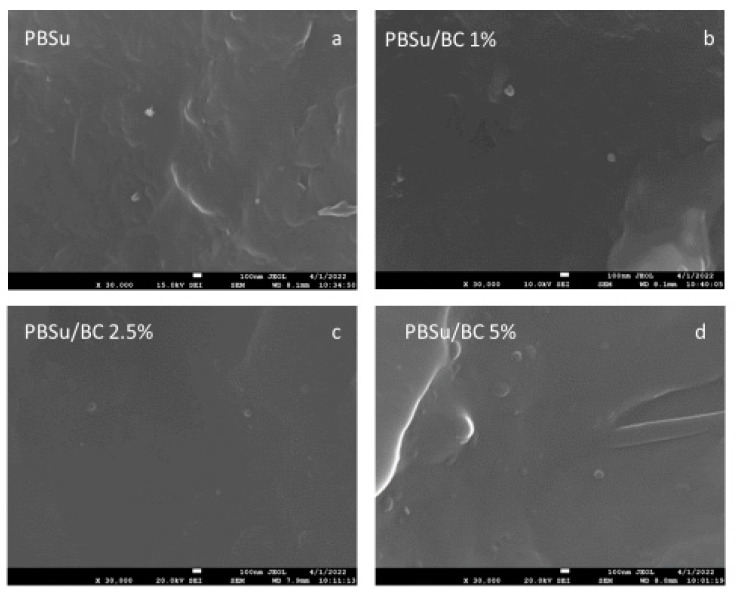
SEM micrographs of the impact fracture surfaces of PBSu/BC composite in high magnification, for samples indicated on the figure.

The physical, mechanical and thermal properties of the materials can affect the surface morphology of the biocomposites. Figure 13 and Figure 14 show the impact fracture surfaces of the PBSu/BC composite under different magnifications. The morphology of the fractured surfaces of the PBSu/BC biocomposites in low magnification suggests good dispersion of BC in the polymer matrix. The appearance of ragged and rough fracture features was observed. These results are in accordance with the study of Luigi Botta, et al. [48]. In particular, all micrographs reveal thin and uniform cracks to the direction of applied force. High magnification micrographs (Figure 14) show that there was no sign of macroscopic aggregation of the BC even at 5 wt% BC content. Also, smooth, homogenous, and uniform fracture surfaces can be seen. There are some spherical particles on the polymers surface, however, it was not clear if these were due to BC since such particles appeared also in micrographs on the neat PBSu. For this reason, all composites have been examined also with TEM. 

### 3.8. BC Dispersion Studied by Transmission Electron Microscopy (TEM)

**Figure 15:**
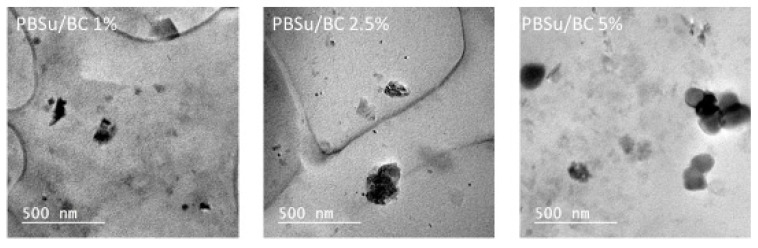
TEM micrographs of PBSu/BC composites with different BC content.

From TEM micrographs taken of all of the composites, it is clear that BC is dispersed in a PBSu matrix in the form of nanosized entities (Figure 15). As can be seen from Figure 15, black, almost spherical particles, with very small sizes of 20–30 nm, and some higher particles with irregular shapes with sizes varied from 130–150 nm, were detected for the sample containing 1 wt% BC. Similar was the appearance to the other composites containing 2.5 and 5 wt% BC, though with particle sizes in the range of 250–300 nm and 400–500 nm, respectively. It seems that as the BC content increases some aggregates were formed, though remaining in the range of nanoparticles. However, from these micrographs, it is clear that even though we started from BC particles with sizes higher than 10 μm, these were able to break down during in situ polymerization at the nanosized level. This fine dispersion of BC nanoparticles is an additional advantage of the polymerization procedure.

### 3.9. Effect of Used BC Filler on PBSu Coloration

The filler content resulted in differences in the coloration of the PBSu biocomposites. The colorimetric coordinates L*, a*, b*, C, h°, and K/S values of the filler content of the biocomposites are given in Table 5. Biocomposites were prepared with BC filler at concentrations 1, 2.5, and 5 wt% and show progressively increasing black coloration from white color (higher L) of pristine PBSu to the blackest material of PBSu/BC 5 wt% (lower L value). These results are in accordance with the work of Mariem Zouari, et al. [19] The K/S fraction, which showed the concentration of the color on the biocomposite increased with the increase of the additive filler. Finally, the PBSu/BC 5 wt% biocomposite possessed a higher concentration of the black color. This is very important for some applications, especially for agricultural mulch films.

### 3.10. Enzymatic Hydrolysis

**Figure 16:**
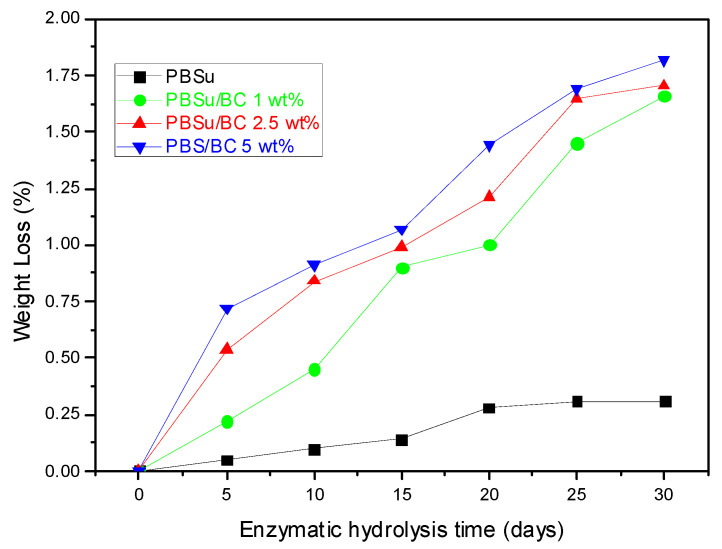
Weight loss vs time plot during enzymatic hydrolysis of neat PBSu and PBSu/BC biocomposites.

PBSu, as it is an aliphatic polyester, has a relatively large number of ester bonds and methylene groups. For this reason, lipases can hydrolyse PBSu and its biocomposites. In this work, the enzymatic hydrolysis of prepared biocomposites took place in an aqueous solution of Rhizopus delemar and Pseudomonas cepacia lipases at 50 °C, due to the low hydrolysis rate of PBSu [49]. The addition of a natural filler, BC, in the PBSu polymeric matrix could result in biocomposite materials that are fully biodegradable. The % weight loss of the samples is presented in Figure 16.

As can be seen, pristine PBSu lost only 0.25% of its initial weight at 30 days of enzymatic hydrolysis. However, all biocomposites had greater weight losses and the rates increased as increasing the BC content. The biocomposites containing 1, 2.5, and 5 wt% BC lost about 1.66, 1.70, and 1.88 wt%, respectively, after 30 days of enzymatic hydrolysis. Thus, it can be concluded that the presence of BC increased the enzymatic hydrolysis rate of PBSu biocomposites, which were easier to decompose by the lipases. The higher enzymatic hydrolysis of the biocomposites can be explained by the creation of bigger voids on their surface, which means that enzymes and water penetrate easier [26,50]. Other parameters that could significantly affect enzymatic hydrolysis are the chemical structure of the polymer, the additive, the amount of the filler, the melting point, the degree of crystallinity, and the molecular weight of the biocomposites [51,52]. However, in our composites, the chemical structure of polyester was the same and the melting points and T_g_ temperatures were identical with neat PBSu, while the degree of crystallinity was slightly higher in the biocomposites (Table 2). The molecular weight was also similar in the neat and the biocomposites. Thus, the effect of all these parameters should be excluded and we can assume that the hydrophilic character of BC was playing the most important role. 

The hydrophilic or hydrophobic character of the filler can strongly affect the degradation rate of enzymatic hydrolysis, as it is associated with the ability of water to penetrate into the biocomposites. The hydrophobicity of the samples was evaluated by measuring their contact angle. As can be seen in Table 6, neat PBSu had a contact angle of 72.67 ± 1.87°. The addition of BC had practically no effect on the hydrophobic nature of the biocomposites with a low BC content since the contact angle of composites with 1 and 2.5 wt% BC content was only slightly decreased until 71.83 ± 1.54° in composite with 2.5 wt% BC. However, at 5 wt% BC, the contact angle was decreased to 69.45 ± 1.59°. A similar decrease was reported in PLA/BC composites [19]. Even though these values were very close to the experimental errors, it can be said that the BC enhances slightly the hydrophilicity of the PBSu polymeric matrix. Thus, the enhanced enzymatic hydrolysis rate of biocomposites should be attributed to this effect as well as to the higher porosity of BC [53] which enhances the ability of water to enter the nanocomposites to a greater extent than in neat PBSu.

**Figure 17:**
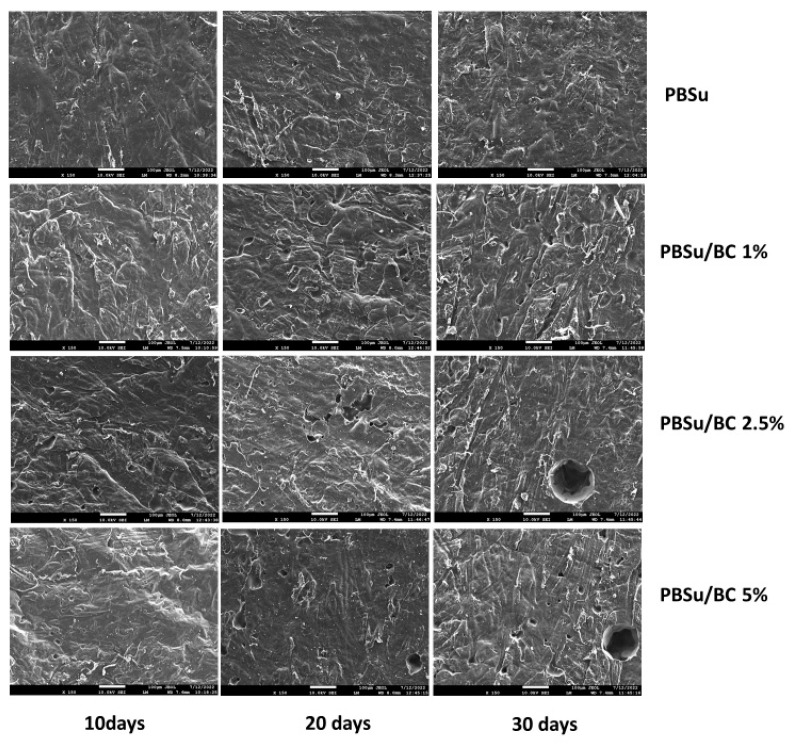
SEM micrographs of PBSu, PBSu/BC 1 wt%, PBSu/BC 2.5 wt%, PBSu/BC 5 wt% during enzymatic hydrolysis at different times (10, 20, and 30 days).

The samples have been studied by SEM to see the surface erosion since enzymatic hydrolysis takes place mainly on the surface of the studied films. In Figure 17 the micrographs presented of PBSu/BC biocomposites at 10, 20, and 30 days of enzymatic hydrolysis, in comparison with neat PBSu.

From pure PBSu micrographs, it was observed that only after 20 days did some very small holes appear, which became more numerous after 30 days. However, in biocomposites, even after 10 days of enzymatic hydrolysis the surfaces of the materials became rougher and holes were observed. After 20 and 30 days of enzymatic hydrolysis, it can be seen that the size and number of the cavities increased, especially in PBSu/BC 2.5 and 5 wt%. These results are in accordance with the weight loss measurements.

## 4. Conclusions

Within this study, fully biodegradable composites of aliphatic polyester PBSu and BC filler at different BC loadings were successfully prepared by in situ polymerization. This method was proven satisfactory in providing good dispersion of the BC in the matrix. GPC verified macromolecules with high *M*_w_ and *M*_n_ values. NMR demonstrated successful synthesis of the PBSu and its PBSu/BC biocomposites while XRD showed that all composites are semicrystalline materials. A fine dispersion of BC throughout the polymer matrix was proven by SEM and TEM micrographs and it was clear that BC was dispersed in nanosized form. From the mechanical study, it was found that the addition of BC increases the tensile strength, Young’s modulus, and impact strength. Regarding BC’s effect on crystallization, its action as a heterogeneous crystallization nucleation agent was confirmed by DSC. Interestingly, the facilitation of crystallization by BC resulted in a noteworthy enhancement of heat transport (thermal diffusivity/conductivity). The influence of the filler on the rate of enzymatic hydrolysis was important, as the BC can reduce the time required for the polymer to degrade. Finally, this work provides basic data for further improving the properties of PBSu biocomposites and expanding the application of PBSu biocomposites as agricultural mulch films or in printing electronics. It would be interesting for further investigation on the thermal stability and the aging effects of the biocomposites.

## Figures and Tables

**Table 1:** Intrinsic viscosity and molecular weight of the prepared PBSu and its biocomposites.

Sample	[η] (dL/g)	Μn (g/mol)	PDI
PBSu	0.89	70,350	1.67
PBSu/BC 1 wt%	0.77	67,080	1.59
PBSu/BC 2.5 wt%	0.85	61,980	1.75
PBSu/BC 5 wt%	0.76	63,730	1.60

**Table 2:** Values of interest by DSC.

	DSC						
Sample	T_c_ (°C)	ΔH_c,n_ (J/g_PBSu_)	CF_c_ (wt) ^&^	T_g_ (°C)	Δ_cp_ (J/g_sample_∙K)	T_cc_ (°C)	T_m_ (°C)
PBSu	64	64	30.47%	−35	0.24	95	115
PBSu/BC 1 wt%	72	69	32.86%	−35	0.24	97	115
PBSu/BC 2.5 wt%	70	70	33.33%	−34	0.23	98	115
PBSu/BC 5 wt%	72	73	34.76%	−34	0.23	98	114

^&^ Estimated by Δ*H*_c,n_ comparing with the heat of fusion Δ*H*_100%cryst_. = 210 J/g for PBSu.

**Table 3:** Values of interest by LFA.

LFA ⁑			
Sample	Ρ (g/cm^3^)	α (mm^2^/s)	λ (W/m∙K)
PBSu	1.41	0.08	0.12
PBSu/BC 1 wt%	1.24	0.11	0.13
PBSu/BC 2.5 wt%	1.21	0.14	0.17
PBSu/BC 5 wt%	1.13	0.20	0.22

⁑ *α* and *λ* values correspond to room temperature.

**Table 4:** Mechanical properties of PBSu/BC biocomposites.

Sample	Tensile Strength at Yield (MPa)	Tensile Strength at Break (MPa)	Elongation at Break (%)	Young’s Modulus (MPa)	Impact Strength (J/m^2^)
PBSu	22.4 ± 0.4	22.2 ± 0.5	440 ± 35	103 ± 8.9	1.29 ± 0.08
PBSu/BC 1 wt%	25.7 ± 0.7	21.2 ± 1.2	131 ± 15	135 ± 4.5	1.53 ± 0.11
PBSu/BC 2.5 wt%	26.7 ± 1.3	26.5 ± 1.5	22 ± 7	144 ± 12	1.74 ± 0.15
PBSu/BC 5 wt%	-	29.0 ± 1.5	20 ± 4	150 ± 11	1.41 ± 0.12

**Table 5:** Colorimetric data L*, a*, b*, C*, h°, and K/S values of the PBSu/BC samples.

Samples	L	a	b	c	h	K/S
PBSu	88.57	−1	3.13	3.03	11.15	0.11
PBSu/BC 1 wt%	29.71	0.05	0.41	0.15	96.93	9.89
PBSu/BC 2.5 wt%	17.39	−0.1	−0.66	0.68	286.95	22.82
PBSu/BC 5 wt%	15.58	0.14	−0.5	0.59	252.32	26.18

**Table 6:** Contact angle measurements of prepared biocomposites.

Sample	Contact Angle (Degree)
PBSu	72.67 ± 1.87
PBSu/BC 1 wt%	70.87 ± 1.11
PBSu/BC 2.5 wt%	71.83 ± 1.54
PBSu/BC 5 wt%	69.45 ± 1.59

## Data Availability

All the data of this study are included in the manuscript.
